# Antitumoral and Antimicrobial Activities of Block Copolymer Micelles Containing Gold Bisdithiolate Complexes

**DOI:** 10.3390/pharmaceutics15020564

**Published:** 2023-02-08

**Authors:** Andreia Sousa, Joana F. Santos, Francisco Silva, Sílvia A. Sousa, Jorge H. Leitão, António P. Matos, Teresa Pinheiro, Rafaela A. L. Silva, Dulce Belo, Manuel Almeida, Fernanda Marques, Célia Fernandes

**Affiliations:** 1 Centro de Ciências e Tecnologias Nucleares, Instituto Superior Técnico, Universidade de Lisboa, Estrada Nacional 10, km 139.7, 2695-066 Bobadela, Portugal; 2 iBB-Institute for Bioengineering and Biosciences and Associate Laboratory Institute for Health and Bioeconomy—i4HB, Instituto Superior Técnico, Universidade de Lisboa, Av. Rovisco Pais, 1049-001 Lisboa, Portugal; 3 Departamento de Bioengenharia, Instituto Superior Técnico, Universidade de Lisboa, Av. Rovisco Pais, 1049-001 Lisboa, Portugal; 4 Centro de Investigação Interdisciplinar Egas Moniz, Campus Universitário, Quinta da Granja, Monte de Caparica, 2829-511 Caparica, Portugal; 5 Departamento de Engenharia e Ciências Nucleares, Instituto Superior Técnico, Universidade de Lisboa, Estrada Nacional 10, km 139.7, 2695-066 Bobadela, Portugal

**Keywords:** block copolymer micelles, drug delivery, gold(III) complexes, antimicrobial, antitumoral

## Abstract

Gold(III) bisdithiolate complexes have been reported as potential antimicrobial and antitumoral agents. The complex [Au(cdc)_2_]^−^ (cdc=cyanodithioimido carbonate) displayed antimicrobial and outstanding antitumor activity against the ovarian cancer cells A2780 and A2780cisR, which are sensitive and resistant to cisplatin, respectively. However, poor water solubility may hamper its clinical use. Block copolymer micelles (BCMs) may solubilize hydrophobic drugs, improving their bioavailability and circulation time in blood. Aiming to provide water solubility, prolonged availability, and enhanced therapeutic indexes, BCMs loaded with [Au(cdc)_2_]^−^ were synthesized and characterized. The BCM-[Au(cdc)_2_] micelles were prepared with a loading efficiency of 64.6% and a loading content of 35.3 mg [Au(cdc)_2_]^−^/gBCM. A hydrodynamic diameter of 77.31 ± 27.00 nm and a low polydispersity index of 0.18 indicated that the micelles were homogenous and good candidates for drug delivery. Cytotoxic activity studies against A2780/A2780cisR cells showed that BCM-[Au(cdc)_2_] maintained relevant cytotoxic activity comparable to the cytotoxicity observed for the same concentration of gold complexes. The Au uptake in A2780 cells, determined by PIXE, was ca. 17% higher for BCMs-[Au(cdc)_2_] compared to [Au(cdc)_2_]^−^. The BCMs-[Au(cdc)_2_] presented antimicrobial activity against *S. aureus* Newman and *C. glabrata* CBS138. These results evidenced the potential of BCM-[Au(cdc)_2_] for drug delivery and its promising anticancer and antimicrobial activities.

## 1. Introduction

One of the main goals of nanomedicine is to develop targeting delivery systems with the ability to reach pathological sites in the body, such as tumors. This strategic approach allows healthy cells to be spared undesired effects, while drug effectiveness and pharmacokinetic profiles can be improved [1]. Nanocarriers, such as polymeric micelles, are appealing drug-delivery systems, particularly for anticancer drugs, due to the reduction in toxicity with the maintenance of therapeutic effects and biocompatibility [2]. These systems can overcome the drawbacks of therapeutic molecules and free drugs, including non-selective activity with respect to targeted tissues, poor water solubility, poor biodistribution and pharmacokinetics (PK), multidrug resistance, dose-limiting toxicity, and fast degradation in vivo [3,4].

Polymeric micelles can be produced from natural polymers or complex synthetic copolymers that have hydrophobic tails and mostly hydrophilic heads—amphiphilic block copolymers. The selection of the polymer has an important role in the formation of micelles, and it is based on the characteristics of both hydrophilic and hydrophobic block polymers. The advantages of polymeric micelles include, among others: (i) an ability to solubilize hydrophobic or poorly water-soluble drugs within their cores, thus enhancing bioavailability; (ii) hydrophilic coronas that allow for longer durations in blood circulation, prevent quick uptake by the reticuloendothelial system (RES), and give steric stability; (iii) biocompatibility; and (iv) low toxicity [3,4,5,6]. 

The delivery of a drug contained in a micelle can be assured by passive or active targeting. Passive targeting exploits pathophysiological and anatomical abnormalities, such as tumor vasculature, and the enhanced permeability and retention (EPR) effect [7]. On the other hand, active targeting exploits the specific interactions (covalent or noncovalent) between polymeric micelles and receptors or antigens on the target cells, which can also promote the internalization of the nanocarriers through receptor-mediated endocytosis. In addition, polymeric micelles can be modified in order to include: (i) surface modification with attached ligands to allow selective targeting and intracellular delivery of drugs; (ii) modifications for “triggered” drug release at diseases sites; (iii) modifications with incorporated dyes for imaging; and (iv) modifications to increase drug life span—an important property for passive targeting [3,7].

Gold and gold compounds have been used for centuries to treat a wide range of diseases, having been found to be effective in treating inflammation, infection, and tuberculosis [8]. In modern times, it has been demonstrated that gold drugs can also be effective in treating rheumatoid arthritis, which culminated in the introduction of the FDA-approved auranofin, a gold(I) triethylphosphine compound [9,10]. However, use of auranofin declined owing to its adverse side effects as a result of its long-standing use in treating chronic diseases. Using a drug-repositioning approach for alternative indications, auranofin is currently being investigated for new therapeutic options, in particular the treatment of some cancers, including leukaemia and ovarian cancer, and parasitic, bacterial, and viral infections [11,12,13].

Several other gold compounds have been designed for pharmacological applications, such as the treatment of cancer and parasitic and viral diseases, although the mechanisms of action are still under investigation. Gold(I) and gold(III) complexes are leaders in prospective medical applications, especially gold(III) complexes [14,15]. Gold (III) complexes display structural similarities to the classical anticancer drug cisplatin (gold(III) is isoelectronic (d8) with platinum(II)). Likewise, they form square planar complexes, and it was therefore expected that such complexes would have similar mechanisms of action. However, extensive reports evidence that gold(III) complexes act by DNA-independent mechanisms, while cisplatin’s mode of action has been linked to its ability to bind to DNA, causing DNA damage [16]. In fact, many studies have evidenced that these gold compounds display high affinities for thiols and selenoproteins and are able to inhibit redox enzymes, such as thioredoxin reductase (TrxR), an effective therapeutic target for cancer, parasitic infections, and HIV [17,18,19]. 

Gold(III) complexes have shown great promise in the treatment of cancer and as antimicrobial agents [20]. Nevertheless, fast hydrolysis and considerable rates of reduction to gold(I) or gold(0) indicate the lability of these complexes. Aliphatic or aromatic nitrogen- or sulphur-donor ligands have the ability to confer stability. On the other hand, their stability can be attained by the careful choice of appropriate inert ligands, such as polydentate ligands with sulphur, oxygen, or nitrogen as electron-donor atoms [21].

**Figure 1:**
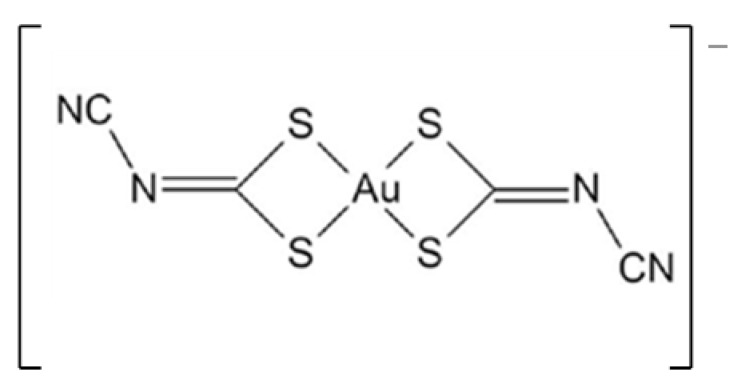
Molecular structure of [Au(cdc)_2_]^−^ (cdc = cyanodithioimido carbonate).

Recently, less explored gold(III) complexes based on bisdithiolenes have emerged as potential therapeutic agents [15,22,23,24,25,26]. One of the candidates, the monoanionic gold bisdithiolate complex TBA[Au(cdc)_2_] (Figure 1) (where cdc = cyanodithioimido carbonate), was screened for its antimicrobial and antitumor activities. The complex presents a significant ability to inhibit the growth of the Gram-positive bacterium *S. aureus* Newman (MIC = 15.3 ± 1.3 μg/mL), the yeast *Candida glabrata* (MIC = 7.0 ± 0.8 µg/mL), and ovarian cancer cells A2780 (IC_50_ = 0.9 ± 0.2 µM) and A2780cisR (IC_50_ = 1.7 ± 0.5 µM), which are sensitive and resistant to cisplatin, respectively. 

However, these complexes have poor solubility in water, which poses some challenges regarding their in vivo administration. The association of these complexes with nanosized carriers, such as micelles, could circumvent the solubility issues and also reduce ligand-displacement reactions with blood proteins [12,27,28,29,30].

The present study reports on the recent advances with TBA[Au(cdc)_2_] (Figure 1) as a new chemotherapeutic drug-delivery agent. This complex has shown important therapeutic properties but exhibits poor solubility in water, like many other chemotherapeutic drugs. The association of these complexes with nanosized carriers, such as polymers and micelles, could improve their solubility, pharmacokinetics, and in vivo circulation times, resulting in increased drug accumulation based on the EPR (enhanced permeability and retention) effect [27,31].

## 2. Materials and Methods

All chemicals and solvents were of reagent grade and used without further purification, unless otherwise stated. Toluene, obtained from Fisher Chemical (Waltham, MA, USA), was dried by distillation with sodium. Dichloromethane was dried with phosphorus pentoxide. Hydrogen chloride solution 2M in diethyl ether, poly(ethylene glycol) methyl ether (Me-PEG, Mn = 5000), and Ɛ-caprolactone (CL) were all acquired from Sigma-Aldrich. Poly(ethylene glycol) methyl ether (Me-PEG, Mn = 5000) was dried twice by azeotropic distillation in toluene, which was distilled off completely, while Ɛ-caprolactone (CL) was dried using calcium hydride and was distilled prior to use. The gold compound TBA[Au(cdc)_2_] was prepared as tetrabutyl ammonium (TBA) salt according to a previously described method [32]. 

The copolymer methoxy-terminated poly(ethylene glycol)-*b*-poly(ε-caprolactone) (Me-PEG-*b*-PCL) was synthesized and characterized following reported synthetic methodologies [33,34] using metal-free cationic ring-opening polymerization of ε-caprolactone (ε-CL) via an activated monomer mechanism with HCl-diethyl ether. UV-Vis spectrophotometry was performed on a Cary 60 UV-Vis spectrophotometer from Agilent Technologies with quartz cuvettes (QS high Precision Cell; 10 mm (Hellma^®^ Analytics, Jena, Germany)), and TBA[Au(cdc)_2_] was quantified with reference to a calibration curve.

High-performance liquid chromatography (HPLC) analysis was performed on a Perkin Elmer Series 200 Pump coupled to a Perkin Elmer Series 200 UV-Vis Detector using as eluents H_2_O with 0.1% of trifluoroacetic acid (TFA) (A) and acetonitrile with 0.1% TFA (B). The eluents were of HPLC grade, and the aqueous solutions were prepared with ultrapure MilliQ water.

TBA[Au(cdc)_2_] analysis was performed in a SUPELCO column (Discovery^®^, Seattle, WA, USA) BIO Wide Pore 300 Å, C18; 25 cm × 4.6 mm, 5 µm (Sigma-Aldrich^®^, St. Louis, MI, USA) at a flow rate of 1 mL/min, using a gradient method: from 60%A/40%B to 10%A/90%B in 15 min. 

### 2.1. Preparation and Characterization of BCMs

**Figure 2:**
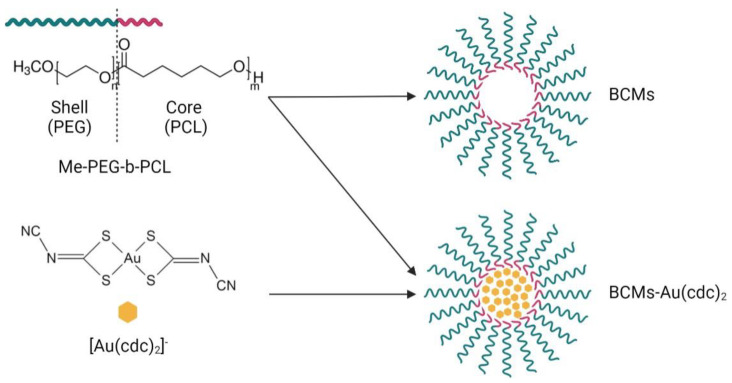
Synthesis of the micelles. BCMs correspond to the non-loaded micelles and BCMs-[Au(cdc)_2_] correspond to the loaded micelles.

BCMs loaded with [Au(cdc)_2_]^−^ were prepared by the thin-film hydration method [33,35]. The polymer (50 mg), Me-PEG-b-PCL, and [Au(cdc)_2_]^−^ (2–8 mg) were dissolved in CHCl_3_ (4 mL) under constant stirring for 4 h at atmospheric pressure and room temperature (RT). Then, the solvent was slowly evaporated overnight under N_2_ flux to form the [Au(cdc)_2_]^−^/ Me-PEG-*b*-PCL thin film, which was hydrated at 60 °C with H_2_O (or PBS) (1 mL) and stirred (low velocity) for 4 h at RT. After the hydration, the solution was centrifuged for 10 min at 1000× *g*, and the supernatant was filtered with a SARTORIUS filter (0.20 µm). The last step of this process was the lyophilization of the micelles. Unloaded micelles were prepared using the same procedure, in the absence of the gold complex (Figure 2).

### 2.2. Drug Loading Content and Efficiency 

**Figure 3:**
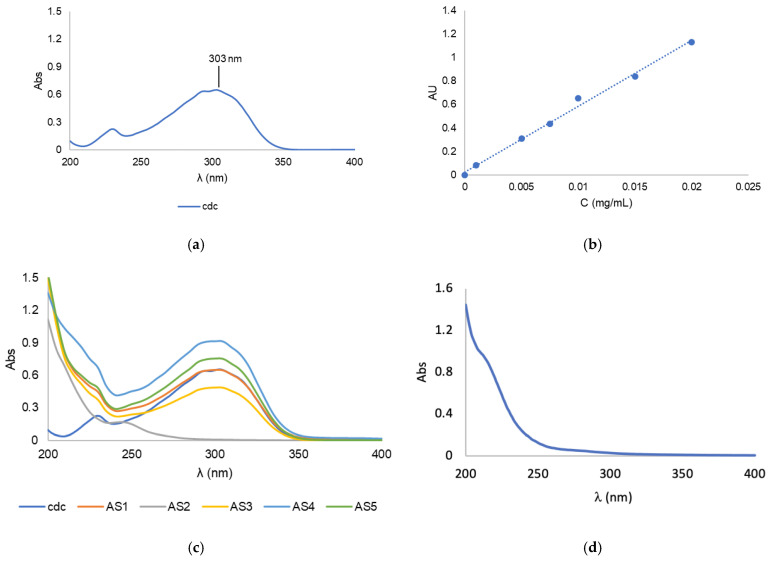
(**a**) UV-vis spectrum of [Au(cdc)_2_]^−^; (**b**) Calibration curve of [Au(cdc)_2_]^−^at *λ* = 303 nm; (**c**) UV-Vis spectra of [Au(cdc)_2_]^−^ and BCMs-[Au(cdc)_2_]; (**d**) UV-vis spectrum of non-loaded BCMs.

The drug loading content (LC) was determined by UV-Vis spectrophotometry (Figure 3) with reference to a standard calibration curve (Figure 3b). For this, 2–4 mg of BCM-Au(cdc)_2_ was dissolved in 1.0 mL of acetonitrile, vortexed and centrifuged at 3000× *g* for 10 min to precipitate the copolymer. The supernatant was then collected and analyzed by UV-Vis spectroscopy.

The [Au(cdc)_2_]^−^ loading content (LC) was calculated using Equation (1):

(1) $$\mathrm{LC}\;(\mathrm{mg}\;{\left[{\mathrm{Au}(\mathrm{cdc})}_{2}\right]}^{-}\;/g_{\mathrm{BCM}}))=\frac{\mathrm{weight}\;\mathrm{of}\;{\left[{\mathrm{Au}(\mathrm{cdc})}_{2}\right]}^{-}\;\mathrm{in}\;\mathrm{the}\;\mathrm{micelles}}{\mathrm{total}\;\mathrm{weight}\;\mathrm{of}\;\mathrm{loaded}\;\mathrm{BCM}\;}$$

The calculation of drug loading efficiency (LE) can be determined using Equation (2):

(2) $$\mathrm{LE}(\%)=\frac{\mathrm{quantitiy}\;\mathrm{of}\;{\left[{\mathrm{Au}(\mathrm{cdc})}_{2}\right]}^{-}\;\mathrm{in}\;\mathrm{the}\;\mathrm{micelles}}{\mathrm{quantity}\;\mathrm{of}\;{\left[{\mathrm{Au}(\mathrm{cdc})}_{2}\right]}^{-}\;\mathrm{used}}\times 100\%$$

### 2.3. Sizes and Zeta Potentials of Micelles

The zeta potentials and hydrodynamic diameters (d_h_s) of the micelles (0.1 g/L) were determined in 0.01 M phosphate buffer (PB) pH 7.4, using a Zetasizer Nano ZS obtained from Malvern with zeta-potential cells. Particle size was measured three times by dynamic light scattering (DLS) at 25 °C with a 173° scattering angle and an optical arrangement known as non-invasive back scatter (NIBS). 

At the beginning of this procedure, the micelles were dissolved in 0.01 M phosphate buffer, pH 7.4 (PB) in order to obtain 1.0 g/L solutions that were subsequently sonicated for 20 min before use. Afterwards, the solutions were diluted and filtered using a 0.20 µm syringe filter. 

### 2.4. Release Study

The in vitro release of [Au(cdc)_2_]^−^ from BCM-Au(cdc)_2_ was evaluated at pH 7.4 using the dialysis method [36,37]. Briefly, a 3 mg solution of BCM-Au(cdc)_2_ in 3 mL of 0.01 M phosphate-buffered saline (PBS) pH 7.4 was placed in a regenerated cellulose tubular dialysis membrane (MWCO = 25 kDa), immersed in 200 mL of 0.01 M PBS pH 7.4, and maintained at 37 °C under continuous stirring. At predetermined time points, 500 µL of the solution inside the dialysis membrane was retrieved and lyophilized, and the membrane was immersed in fresh medium. Afterwards, 500 µL of acetonitrile was added to the retrieved solutions, and the resultant solutions were vortexed and centrifuged at 3000× *g* for 10 min to precipitate the copolymer and the PBS salts. The supernatant was collected and analyzed by UV-vis spectrophotometry. The drug-release profile was calculated as the cumulative percentage of released [Au(cdc)_2_]^−^ over time, and 100% release corresponds to the total amount of [Au(cdc)_2_]^−^ entrapped in the micelles.

### 2.5. Cells and Cell-Culture Media

A2780 (cisplatin-sensitive) and A2780cisR (cisplatin-resistant) ovarian tumor cells were purchased from Sigma-Aldrich. V79 (hamster lung fibroblasts) were purchased from the ATCC (American Type Culture Collection). Cell media and media supplements were purchased from Gibco (Thermo Fisher Scientific). All cell lines were grown in RPMI medium (Gibco) supplemented with 10% FBS (Gibco) and maintained in a humidified incubator (Heraeus, Hanau, Germany) with 5% CO_2_.

### 2.6. Determination of Cytotoxic Activity 

The cytotoxic activities of BCMs, BCMs-[Au(cdc)_2_]^−^, and [Au(cdc)_2_]^−^ were evaluated with the matched pair of cisplatin-sensitive/-resistant A2780 cell lines (A2780/A2780cisR) and in normal fibroblasts (V79), using the MTT assay, as previously described [25]. For the assays, cells (1–2 × 10^4^ cells/200 μL medium) were seeded in 96-well plates and allowed to adhere for 24 h. Loaded BCMs (drug loading content: 3.56%) were diluted to prepare serial concentrations in the range of 10 ng/mL–2 g/mL—concentrations that correspond to 10^−7^–10^−4^ M of [Au(cdc)_2_]^−^. Unloaded BCMs were diluted in medium to prepare serial dilutions in the range of 10 ng/mL–2 g/mL. Loaded and unloaded BCMs were added to the cells and incubated for 48 h at 37 °C. At the end of the treatment, the MTT assay was used, following a procedure similar to one previously described [25].

### 2.7. Cellular Uptake Analysis

The concentration of gold in A2780 cell pellets after incubation with [Au(cdc)_2_]^−^ or BCM-[Au(cdc)_2_] was determined by particle-induced X-ray emission (PIXE), installed at the Van de Graaff accelerator of the Centro Tecnológico e Nuclear, Instituto Superior Técnico. A2780 cells were incubated with [Au(cdc)_2_]^−^ and BCM-[Au(cdc)_2_] at 9.0 µM, and the IC_50_ values were determined after 3 h incubation. The cell pellets were obtained by centrifugation after washing of the cells with PBS to remove the medium. The samples were freeze-dried and microwave-assisted acid-digested in aqua regia with yttrium as an internal standard, as previously described [26]. The concentrations of Au in the cell pellets were obtained in µg/g dry weight and converted to ng/10^6^ cells.

### 2.8. Antimicrobial Activities of BCMs

The antimicrobial activities of the BCMs and BCMs-Au(cdc)_2_ towards *S. aureus* Newman and the pathogenic fungi *Candida glabrata* CBS138 were assessed by the determination of minimum inhibitory concentrations (MICs) using microdilution assays, as previously described [25] and according to EUCAST (European Committee on Antimicrobial Susceptibility Testing) recommendations [38,39]. Both strains were isolated from human infections [40,41]. *S. aureus* Newman was maintained in Lennox Broth (LB) solid medium, composed of 10 g/L tryptone, 5 g/L yeast extract, 5 g/L NaCl, and 20 g/L agar. *C. glabrata* CBS138 was maintained in yeast extract–peptone–dextrose (YPD) solid medium (20 g/L glucose, 20 g/L peptone, 10 g/L yeast extract, and 15 g/L agar).

Briefly, stock solutions of the BCMs and BCMs-Au(cdc)_2_ were prepared in Mueller–Hinton (MH) broth (Sigma-Aldrich) (for *S. aureus*) or RPMI-1640 liquid medium (Sigma) buffered to pH 7.0 with 0.165 M morpholinepropanesulphonic acid (MOPS, Sigma) (for *C. glabrata*) at final concentrations of 5 mg/mL (180 µg/mL of [Au(cdc)_2_]^−^). Serial 1:2 dilutions of stock solutions were prepared for each micelle preparation in MH broth or RPMI-1640, the final concentrations being between 1200 and 4.69 μg/mL (45 and 0.17 μg/mL of [Au(cdc)_2_]^−^). 

Then, for *S. aureus*, 100 μL aliquots of adequately diluted bacterial suspensions were mixed with the MH broth serially diluted micelle aliquots to obtain $5\times 10^{5}$ CFU/mL. Bacterial suspensions were prepared from cultures grown for 5 h in MH broth at 37 °C and 250 rev·min^−1^ and adequately diluted with fresh MH broth. After 22 h of incubation at 37 °C, the well contents were resuspended by pipetting, and the optical densities were measured in a SPECTROstar Nano microplate reader (BMG Labtech) at 640 nm.

*C. glabrata* overnight-grown fungal cultures (carried out in YPD broth at 30 °C and 250 rev. min^−1^) were diluted with fresh RPMI-1640 liquid medium to a final optical density of 0.025, measured at 530 nm (OD_530_) using a Hitachi U-2000 UV/Vis spectrophotometer. The wells were then inoculated with the addition of 100 μL of fungal suspensions and incubated for 24 h at 35 °C. After incubation, the wells were examined for turbidity (growth) and resuspended, and their optical densities were measured using a SPECTROstar Nano microplate reader (BMG Labtech) at 530 nm.

At least two independent experiments were performed in duplicate for each micelle preparation under study. The minimum inhibitory concentration (MIC) was defined as the lowest concentration of the antimicrobial that inhibited the visible growth of a microorganism after the incubation time. Positive (no micelles) and negative controls (no inoculum) were performed for each experiment. Micelles at the same concentrations used in the assay without inoculum were also tested, as negative controls.

## 3. Results and Discussion

### 3.1. Synthesis and Characterization of Block Copolymer Micelles

The block copolymer micelles (BCMs) prepared in this work were synthesized according to the thin-film hydration method [33,35]. The [Au(cdc)_2_]^−^ loaded and non-loaded micelles, BCMs-Au(cdc)_2_ and BCMs, were formed by self-assembly using Me-PEG-b-PCL (Figure 2), an amphiphilic polymer with polyethylene glycol (PEG) as the hydrophilic chain and polycaprolactone (PCL) as the hydrophobic chain. Due to the low water solubility of [Au(cdc)_2_]^−^, the gold complexes are encapsulated inside the micelles when the self-assembly of the polymer occurs in aqueous solution. 

To ensure an optimal loading content of the [Au(cdc)_2_]^−^ gold complexes in the BCMs, various parameters were evaluated. In the first part of the optimization process, the influences of different types of solvents used for the dissolution of the polymer and the gold complex and for the hydration of the thin film were studied (Table 1). For the dissolution of the polymer and [Au(cdc)_2_]^−^, CHCl_3_, ACN, or a mixture of DMF with DCM were used, while hydration was evaluated using H_2_O or PBS. The amounts of [Au(cdc)_2_]^−^ and polymer used were maintained at 2 mg and 25 mg, respectively. In addition, volumes of solvents for the dissolution ranged between 2 and 4 mL, and 1 mL for the solvent was used in the hydration of the thin film. 

In order to assess the loading contents of the resulting BCMs-[Au(cdc)_2_] (formulations AS1–AS5), known amounts of the micelles were disassembled using acetonitrile followed by UV-Vis analysis of the solubilized contents (Figure 3). The results were compared with a standard calibration curve (Figure 3b) previously obtained using solutions of known concentrations of [Au(cdc)_2_]^−^, with values for the maximum absorbance of the gold complexes acquired at 303 nm (Figure 3a). 

The results indicated a clear influence of the type of solvent used on LC, both for the polymer and gold-complex solubilization, as well as for the thin-film hydration, as can be seen in Table 2. Using ACN as a solubilization agent led to the lowest [Au(cdc)_2_]^−^ LC results; in fact, in the case of formulation AS2, no gold-complex encapsulation was obtained. In contrast, CHCl_3_ led to the highest amount of loaded [Au(cdc)_2_]^−^, as evidenced by the results obtained for formulations AS4 and AS5. Regarding the thin-film hydration solvent used, a higher loading was obtained using H_2_O compared to PBS. Water led to the micelle formulation AS4 standing out as the one with the highest [Au(cdc)_2_]^−^ loading content (LC (mg_[Au(cdc)2]−/_g_BCM_) = 5.68). The variations in LCs obtained using water or PBS were not as significant as those obtained with the change of the polymer and the gold-complex solubilization solvent.

Aiming at the optimization of the [Au(cdc)_2_]^−^ loading process, we then studied the impact of the drug/copolymer ratio. For this purpose, we used a constant amount of copolymer (50 mg) and amounts of gold complexes in the range of 1–8 mg (Table 3).

**Figure 4:**
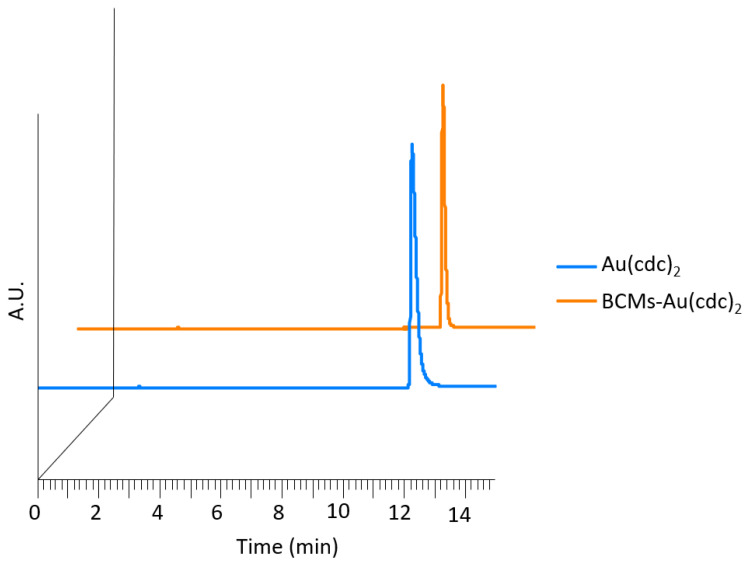
HPLC chromatograms of TBA[Au(cdc)_2_] (R*_t_*= 12.3 min) and gold complexes released from the BCMs-[Au(cdc)_2_] (R*_t_ =* 12.3 min) (UV detection at *λ* = 303 nm).

The results presented in Table 3 show that the drug loading contents and efficiencies were higher for lower quantities of [Au(cdc)_2_]^−^, with the highest loading content being found for the micelle formulation AS6 (LC = 35.29 mg_[Au(cdc)2]^−^_/g_BCM_). The structural integrity of the gold complex [Au(cdc)_2_]^−^ upon encapsulation in the micelles was investigated by high-performance liquid chromatography (HPLC) analysis. After disassembly of the loaded micelles, the supernatants were analyzed by HPLC and the results were compared to those of the gold complexes before encapsulation (Figure 4). The HPLC chromatograms show that the free gold complexes and the gold complexes collected from the loaded micelles exhibited similar profiles, with single peaks at similar retention times, suggesting that the gold complexes maintained their chemical structures unaltered.

The drug loading content reported for auranofin-loaded nanoparticles was in the range of 1.8–6.2 mg of auranofin per gram of NPs [42] as compared to 35.29 mg of [Au(cdc)_2_]^−^ per gram of micelles for formulation AS6. These results indicate that [Au(cdc)_2_]^−^ is encapsulated more efficiently than auranofin using this optimized formulation, which is a remarkable result for these newly developed BCMs. 

The hydrodynamic diameters (d_h_s) and the zeta potentials (Z_p_s) of the micelles were determined by DLS and LDV, respectively. After sample dilution with PBS (pH = 7.4, 0.01 M), measurements were carried out, and the values obtained for each sample are presented in Table 4. This study was conducted for the micelle formulations AS1, AS4, and AS6. 

The morphology of the micelles (BCMs and BCMs-[Au(cdc)_2_]) was determined by transmission electron microscopy (TEM) (Figure S2).

For a long circulation half-life, polymeric micelles should have a hydrodynamic diameter in the range of 10–100 nm [4]. The micelles studied herein exhibited hydrodynamic diameters within this range. However, the differences in the LCs for samples A1, A4, and A6 did not translate into significant variations in d_h_ values. This may have been due to the planar molecular geometries of the gold complexes, which, at higher concentrations, may force the complexes into a uniform stacking, instead of a random spatial orientation, favourable at lower concentrations. Nevertheless, the results also showed that the hydrodynamic diameters (d_h_s) were higher for micelles with higher loading contents (77.31 ± 27.00 nm for AS6). The polydispersity indexes (PdIs) suggest that the samples were homogeneous, with values comparable to those for other micelles synthesized from the copolymer PEG-*b*-PCL [33,43,44]. 

Absolute zeta potential (Z_p_) values of >30 mV are required for full electrostatic stabilization. Z_p_ values within the range of 5–15 mV are in the region of limited flocculation, while micelles with Z_p_ values lower than 3 mV will exhibit a maximal tendency to flocculate. Hence, particle aggregation is less expected to occur for charged particles (high Z_p_) due to electric repulsion. The micelles studied exhibited absolute Z_p_ values > 50 mV, indicative of their suitable stability in aqueous media and low tendency for aggregation. 

**Figure 5:**
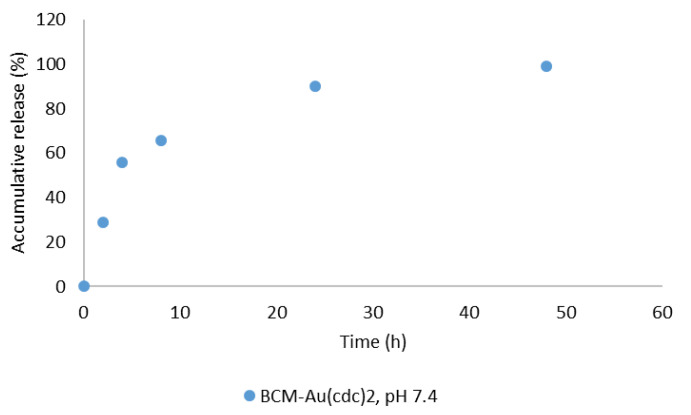
In vitro [Au(cdc)_2_]^−^ release profile for BCM-[Au(cdc)_2_] at pH 7.4.

In vitro release studies were performed at different time points using the AS6 micelles at pH 7.4 and 37 °C, using the dialysis method [36,37]. The results obtained (Figure 5) showed a steady and controlled release of the gold complexes from the micelles. At 24 h post incubation, about 89% of the [Au(cdc)_2_]^−^ was already released from the BCM structures. 

### 3.2. Biological Studies

Given the highest LC observed for AS6, as well as the suitable d_h_ and Z_p_ values displayed by this formulation, these micelles were selected for in vitro biological studies. Hence, the results reported below are based on the AS6 BCMs.

#### 3.2.1. Cytotoxic Activity

**Figure 6:**
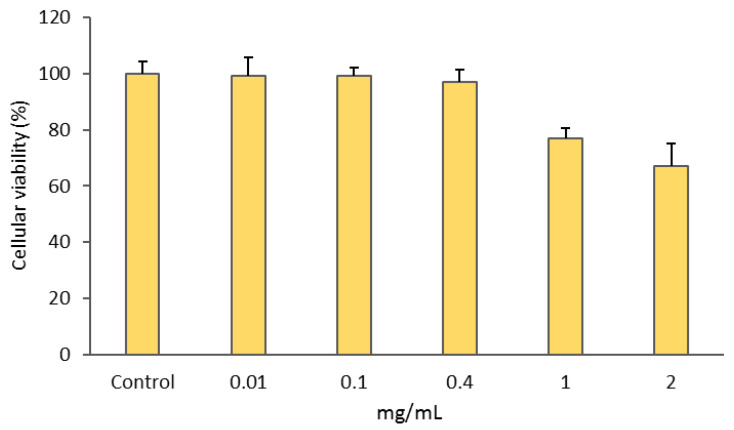
Cellular viabilities of A2780 cells after incubation with BCMs (unloaded micelles) for 48 h. Results shown are the means ± SDs of at least two experiments performed with six replicates each.

The gold complex [Au(cdc)_2_]^−^ was previously shown to exhibit a remarkable antiproliferative activity towards A2780 ovarian cancer cells [25]. Herein, we evaluated the retention of [Au(cdc)_2_]^−^ activity upon encapsulation in the BCMs. The MTT assay was used to compare the activities of [Au(cdc)_2_]^−^ and BCM-Au(cdc)_2_ in the ovarian cancer cells A2780 and A2780cisR and normal V79 fibroblasts (Table 5, Figure S1). Unloaded BCMs were also included as controls in order to confirm whether the cytotoxic activity exhibited by BCM-[Au(cdc)_2_] was due to the gold complexes and not the unloaded micelles (Figure 6). 

The results presented in Table 5 show that the IC_50_ values obtained for BCM-[Au(cdc)_2_] are similar to those of [Au(cdc)_2_]^−^ for each cancer cell line and the normal fibroblasts. The V79 cells were included to ascertain the selectivity of [Au(cdc)_2_]^−^ and BCM-[Au(cdc)_2_] for cancer cells. The low IC_50_ values of BCMs-Au(cdc)_2_ for both sensitive and resistant ovarian cancer cells (1.83 vs. 1.69), as well as the selectivity index (SI) values for both compounds in these cells (SI > 2), suggest that these compounds are promising therapeutic agents for ovarian cancer and better candidates when compared with auranofin, the reference drug, which has a lower SI value (<2) [25,45].

Additionally, the BCMs without encapsulated gold complexes (Figure 6) displayed a ca. 30% loss of cellular viability only at the highest concentration of 2 mg/mL. 

Overall, these results show that [Au(cdc)_2_]^−^ is still able to maintain its cytotoxic activity after micelle encapsulation. Moreover, the results are also in agreement with others found for the reference drug auranofin. In fact, this gold(I) complex encapsulated in a micellar form (a block copolymer system) displayed a similar activity against ovarian cancer cells (OVCAR3) when compared with the complex itself. Using this micellar platform, the instability of auranofin was overcome due to the presence of protein thiols and their unspecific toxicity [30].

#### 3.2.2. Quantification of Cellular Uptake of Free and Encapsulated [Au(cdc)_2_]^−^ by PIXE

Since it is assumed that the therapeutic effect could depend on the amount of gold complexes within cells, the net uptake of both [Au(cdc)_2_]^−^ and BCM-[Au(cdc)_2_] in whole A2780 cells was evaluated using the PIXE technique (Table 6). 

For these studies, the IC_50_ values at shorter incubation times (3 h) were determined as part of a compromise to ensure that the compounds entered the cells and deposited measurable Au levels. The PIXE technique offers advantages in assessing Au contents in cell extracts due to its high sensitivity (in the ppm range) and accuracy in determining elemental concentrations, even with small sample masses. The estimated detection limit for Au < 30 μg/g dry weight was two orders of magnitude below the Au concentration in the sample, and the precision of Au determination was below 5%.

The results presented in Table 6 were obtained after incubating the cells for 3 h with the compounds at their IC_50_ values. The Au uptake by A2780 treated cells was ca. 17% higher for BCM-[Au(cdc)_2_] compared to [Au(cdc)_2_]^−^, which indicates that the rate of uptake of the loaded micelles was faster.

#### 3.2.3. Antimicrobial Activities

The antimicrobial properties of the BCMs and BCMs-[Au(cdc)_2_]^−^ were assessed based on the determination of the MIC values with respect to the Gram-positive bacteria *S. aureus* Newman and the yeast *C. glabrata* CBS138, using the microdilution method (Table 7). 

The BCMs-[Au(cdc)_2_]^−^ presented antimicrobial activity against *S. aureus* Newman and *C. glabrata* CBS138. As expected, BCMs not loaded with [Au(cdc)_2_]^−^ had no antimicrobial activity.

## 4. Conclusions

Polymeric micelles have emerged as alternatives to deliver therapeutic drugs more selectively, improve bioavailability, enhance drug release, and maintain therapeutic drug plasma levels, with reduced side effects. In addition, BCMs are able to increase drug solubility and stability and control delivery rates. In particular, block copolymer micelles have demonstrated great potential as nanocarriers capable of delivering hydrophobic drugs and controlling their distribution and function. The formulation of such delivery nanocarriers requires reliable characterization and evaluation of their efficacy at a biological level. This paper outlines our recent studies on block copolymer micelles to deliver prospective metal-based complexes with antitumoral and antimicrobial activities. 

To sum up, BCMs prepared with Me-PEG-b-PCL chains, loaded and non-loaded with gold bisdithiolate complexes ([Au(cdc)_2_]^−^), were successfully synthesized. The hydrodynamic diameters were below 100 nm, and the zeta potential values were indicative of high stability and a low tendency to form aggregates. The synthesis of the micelles was optimized to achieve a high loading content of [Au(cdc)_2_]^−^ (35.29 mg[Au(cdc)_2_]^−^/gBCM). Additionally, HPLC analysis and UV-vis spectrophotometry data indicated that the gold complexes maintained their stability after encapsulation in the micelles. The loaded micelles displayed significant cytotoxic activity towards the ovarian pair A2780/A2780cisR and equivalent activity for both cell lines. Compared with the reference drug auranofin, the BCMs-[Au(cdc)_2_]^−^ displayed a better profile in terms of general cytotoxicity, i.e., a higher SI, when cancer cells were compared with normal fibroblasts. The results showed that, after micelle encapsulation, [Au(cdc)_2_]^−^ was still able to maintain its cytotoxic activity against ovarian cancer cells and its antimicrobial activity against *S. aureus* Newman and *glabrata* CBS138. 

Overall, the results obtained evidenced the potential of BCM-[Au(cdc)_2_] as a novel drug-delivery system with promising anticancer and antimicrobial activities that deserve to be further evaluated. 

## Figures and Tables

**Table 1:** Formulations used to optimize the formation of micelles loaded with [Au(cdc)_2_]^−^.

	Complex (mg)	Polymer (mg)	Solvents for Complex Dissolution	Solvents for Thin-Film Hydration
**AS1**	2	25	DMF + DCM	PBS
**AS2**			ACN	H_2_O
**AS3**			ACN	PBS
**AS4**			CHCl_3_	H_2_O
**AS5**			CHCl_3_	PBS

**Table 2:** Loading contents (LCs) and loading efficiencies (LEs) of the resulting BCMs-[Au(cdc)_2_] (formulations AS1–AS5).

	m_total Au(cdc)_ (mg)	m_BCM_ (mg)	LE (%)	LC (mg_cdc_/g_BCM_)
**AS1**	0.145	34.5	7.3	4.21
**AS2**	0.000	5.9	0.0	0.00
**AS3**	0.070	22.8	3.5	3.05
**AS4**	0.096	16.9	4.8	5.68
**AS5**	0.118	22.4	5.9	5.27

**Table 3:** Loading efficiencies (LEs) and loading contents (LCs) for the indicated amounts of gold complexes (m_cdc_).

	m_Au(cdc)_ (mg)	LE (%)	LC (mg_[Au(cdc)2]^−^/_g_BCM_)
**AS6**	2	64.59	35.29
**AS7**	4	7.81	8.47
**AS8**	8	3.34	7.11
**AS9**	1	65.68	24.25

**Table 4:** Hydrodynamic diameters (d_h_s), polydispersions (PdIs), and zeta potentials (Z_p_s) of micelle formulations AS1, AS4, and AS6.

	d_h_ (nm)	PdI	Z_p_ (mV)
**AS1**	50.96 ± 20.06	0.42	−63.03 ± 9.75
**AS4**	70.56 ± 35.49	0.30	−51.10 ± 9.92
**AS6**	77.31 ± 27.00	0.18	−57.20 ± 12.10

**Table 5:** The cytotoxic activities of [Au(cdc)_2_]^−^ and BCM-[Au(cdc)_2_] after 48 h of incubation according to the MTT assay. Results are presented as the means ± SDs of at least two experiments performed with six replicates per condition.

Compound	IC_50_ (µM)		
	A2780	A2780cisR	V79
**[Au(cdc)_2_]^−^**	1.11 ± 0.24	1.63 ± 0.41	3.36 ± 0.92
**BCM-[Au(cdc)_2_]**	1.83 ± 0.62	1.69 ± 0.43	3.65 ± 0.95

**Table 6:** Cellular uptake of Au in whole A2780 cells as determined by PIXE. Cells were previously incubated with the compounds at 9 μM for 3 h. The Au levels in the whole cells are expressed as ng Au/10^6^ cells.

Compound	Celular Uptake ng Au/10^6^ A2780 Cells
**[Au(cdc)_2_]^−^**	321 ± 16
**BCM-[Au(cdc)_2_]**	387 ± 13

**Table 7:** Estimated minimum inhibitory concentrations (MICs) of BCMs-[Au(cdc)_2_]^−^ and BCMs with respect to *S. aureus* Newman and *C. glabrata* CBS138. Results are the means of two independent experiments performed with two replicates. MIC values were estimated considering the total amounts of BCMs-[Au(cdc)_2_]^−^ (Total) or the amounts of [Au(cdc)_2_]^−^ encapsulated in BCMs ([Au(cdc)_2_]^−^ Amount).

	MIC (µg/mL)			
	BCMs-[Au(cdc)_2_]^−^		BCMs	Free [Au(cdc)_2_]^−^
	Total	[Au(cdc)_2_]^−^ Amount		
***S. aureus* Newman**	498.9 ± 142.9	18.7 ± 5.4	>1250	15.3 ± 1.3 *
***C. glabrata* CBS138**	490.5 ± 241.8	18.9 ± 8.4	>1250	7.0 ± 0.8 *

* Values taken from [25].

## Data Availability

Not applicable.

## Supplementary Materials

The following supporting information can be downloaded at: https://www.mdpi.com/article/10.3390/pharmaceutics15020564/s1, Figure S1: Dose–response curves to determine IC_50_ values using GraphPad Prism software (vs 5.0); Figure S2: Characterization of BCMs and BCMs-[Au(cdc)2] by TEM.
