# Supplementary material for: Antitumoral and Antimicrobial Activities of Block Copolymer Micelles Containing Gold Bisdithiolate Complexes
Source: Pharmaceutics. 2023 Feb 8;15(2):564. doi: 10.3390/pharmaceutics15020564 (PMC9964654; doi:10.3390/pharmaceutics15020564)
Supplement: Supplementary file 1 [file pharmaceutics-15-00564-s001.zip › pharmaceutics-2177908-supplementary.pdf]

Antitumoral and antimicrobial activities of block copolymer micelles containing a gold bisdithiolate complex

Andreia Sousa[1], Joana F. Santos[1], Francisco Silva[1#], Sílvia A. Sousa[2,3], Jorge H. Leitão[2,3], António P. Matos[4], Teresa Pinheiro[2,5], Rafaela A. L. Silva[1,5], Dulce Belo[1,5], Manuel Almeida[1,5], Fernanda Marques[1,5]*, Célia Fernandes[1,5]

[1]Centro de Ciências e Tecnologias Nucleares, Instituto Superior Técnico, Universidade de Lisboa, Estrada Nacional 10, km 139.7, 2695-066 Bobadela LRS, Portugal.

[2]iBB-Institute for Bioengineering and Biosciences and Associate Laboratory Institute for Health and Bioeconomy—i4HB at Instituto Superior Técnico, Av. Rovisco Pais, 1049-001 Lisboa, Portugal.

[3] Departamento de Bioengenharia, Instituto Superior Técnico, Universidade de Lisboa, Av. Rovisco Pais, 1049-001 Lisboa, Portugal.

[4]Centro de Investigação Interdisciplinar Egas Moniz, Campus Universitário, Quinta da Granja, Monte de Caparica, 2829-511 Caparica, Portugal.

[5]Departamento de Engenharia e Ciências Nucleares, Instituto Superior Técnico, Universidade de Lisboa, Estrada Nacional 10, km 139.7, 2695-066 Bobadela LRS, Portugal.

[#] Present address: Champalimaud Centre for the Unknown, Fundação Champalimaud, Av. Brasília, 1400-038 Lisboa.

Table of Contents

Figure S1. **2-3**

Figure S2. **3**

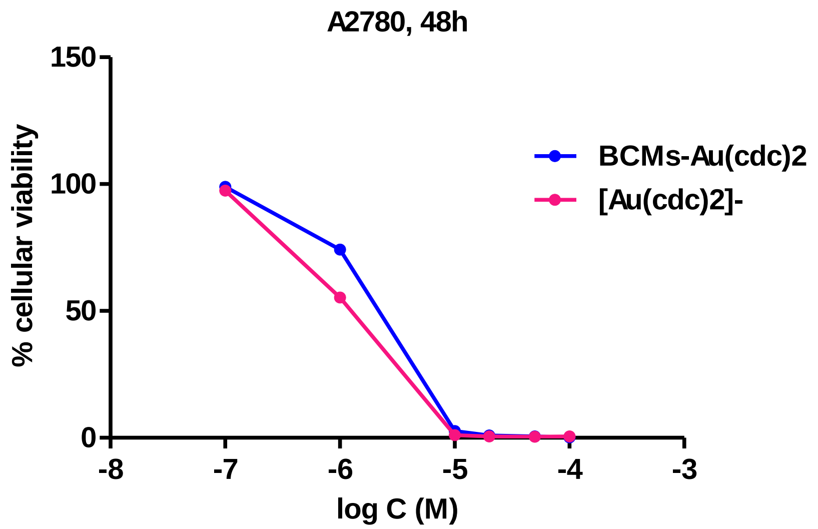
A2780, 48h
150
100
50
0
% cellular viability
BCMs-Au(cdc)2
[Au(cdc)2]-
-8
-7
-6
-5
-4
-3
log C (M)

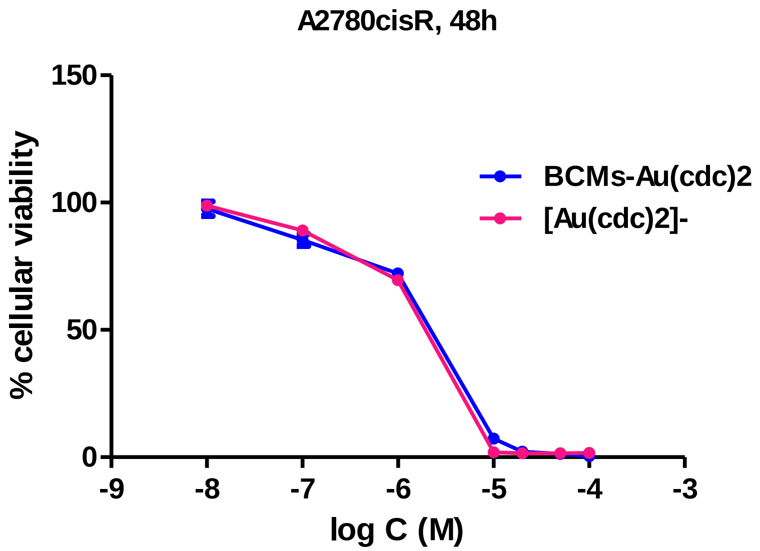
A2780cisR, 48h
150
100
50
0
% cellular viability
BCMs-Au(cdc)2
[Au(cdc)2]-
-9
-8
-7
-6
-5
-4
-3
log C (M)

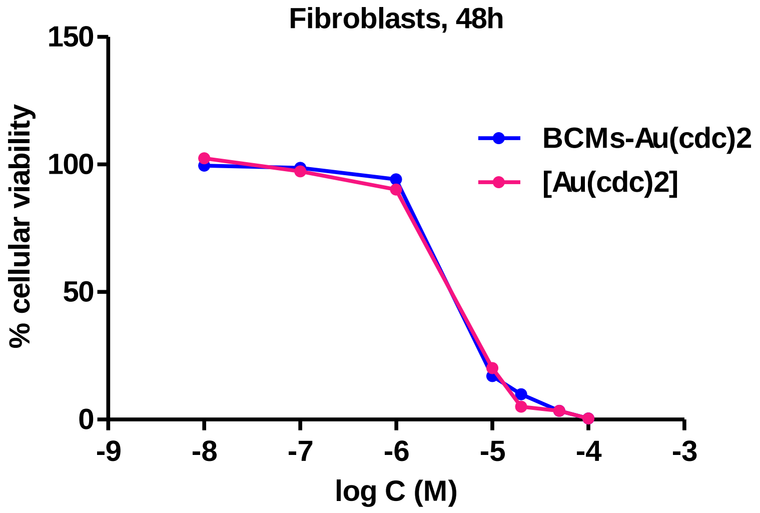

Figure S1. Dose-response curves to determine the IC_{50} values using the GraphPad Prism software (*vs* 5.0).

Transmission electron microscopy (TEM)

The morphology of the micelles was evaluated by transmission electron microscopy (TEM). The non-loaded BCMs were dissolved in 0.01 M PB, pH 7.4 at 0.1 g/L and were placed on negatively charged carbon-coated copper grids, left to dry at RT and observed by transmission electron microscopy (TEM) with a Hitachi H-8100 electron microscope operating at 500 keV. An aliquot of BMCs-Au(cdc)2 was adsorbed onto formvar-carbon coated grids and stained with 2% aqueous uranyl acetate for 2 minutes. Excess staining was drained with filter paper and the grids were air dried before being observed and photographed in a JEOL 1200EX transmission electron microscope.

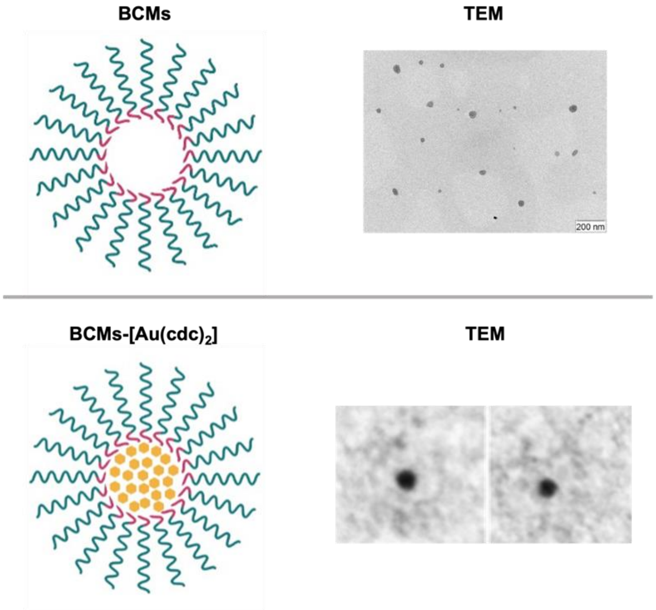

Figure S2. Characterization of BCMs and BCMs-[Au(cdc)2] by TEM.
